# An Enhanced Pedestrian Visual-Inertial SLAM System Aided with Vanishing Point in Indoor Environments

**DOI:** 10.3390/s21227428

**Published:** 2021-11-09

**Authors:** Wennan Chai, Chao Li, Mingyue Zhang, Zhen Sun, Hao Yuan, Fanyu Lin, Qingdang Li

**Affiliations:** 1 College of Sino-German Science and Technology, Qingdao University of Science and Technology, Qingdao 266061, China; chaiwennan@qust.edu.cn (W.C.); zin1121@qust.edu.cn (M.Z.); linfy-cdtf@qust.edu.cn (F.L.); 2 College of Automation and Electronic Engineering, Qingdao University of Science and Technology, Qingdao 266061, China; lichao_1109@mails.qust.edu.cn (C.L.); yuanhao@mails.qust.edu.cn (H.Y.); 3 College of Information Science and Technology, Qingdao University of Science and Technology, Qingdao 266061, China; sunzhen@qust.edu.cn

**Keywords:** simultaneous localization and mapping, inertial navigation system, vanishing point, local optimization, global optimization, pedestrian dead reckoning

## Abstract

The visual-inertial simultaneous localization and mapping (SLAM) is a feasible indoor positioning system that combines the visual SLAM with inertial navigation. There are accumulated drift errors in inertial navigation due to the state propagation and the bias of the inertial measurement unit (IMU) sensor. The visual-inertial SLAM can correct the drift errors via loop detection and local pose optimization. However, if the trajectory is not a closed loop, the drift error might not be significantly reduced. This paper presents a novel pedestrian dead reckoning (PDR)-aided visual-inertial SLAM, taking advantage of the enhanced vanishing point (VP) observation. The VP is integrated into the visual-inertial SLAM as an external observation without drift error to correct the system drift error. Additionally, the estimated trajectory’s scale is affected by the IMU measurement errors in visual-inertial SLAM. Pedestrian dead reckoning (PDR) velocity is employed to constrain the double integration result of acceleration measurement from the IMU. Furthermore, to enhance the proposed system’s robustness and the positioning accuracy, the local optimization based on the sliding window and the global optimization based on the segmentation window are proposed. A series of experiments are conducted using the public ADVIO dataset and a self-collected dataset to compare the proposed system with the visual-inertial SLAM. Finally, the results demonstrate that the proposed optimization method can effectively correct the accumulated drift error in the proposed visual-inertial SLAM system.

## 1. Introduction

Due to the complementary characteristics of the camera and the IMU, visual-inertial SLAM has become a hot topic. Additionally, the system has a wide range of potential applications in smartphones, robots, autonomous driving, and augmented reality (AR) [1,2,3,4]. Many scholars have attempted to improve the positioning accuracy and robustness of the visual-inertial SLAM under various conditions [5,6]. However, the long-term drift is unavoidable for visual-inertial SLAM without loop closures, especially when the trajectory is long [7]. Loop closures detection, which refers to a graph optimization method, is used to correct drift error [6,7]. To detect loop closures, a similar viewpoint for feature point matching is required. Additionally, the end of the trajectory must exceed the starting point of the trajectory by a specified distance to avoid any loop closure detection failures caused by system initialization. Therefore, detecting loop closures in application scenes, particularly in indoor environments, is challenging. Hence, an external observation without drift error is required to correct system drift error. However, there are few global observations without drift error in indoor environments, especially observations that can be extracted from images.

The VP has attracted considerable attention since its inception and has been used in a wide variety of applications, including camera calibration and relative pose estimation [8]. The following are the key advantages of external observation VP: (1) the attitude estimation based on VP has no drift error, which can be used to correct the system drift error; (2) because VP detection does not require continuity, the system can adjust the detection frame rate flexibly in accordance with available computing power; (3) the VP detection is based on image perspective principles, which means that the system requires no additional hardware. Similarly, the experimentation demonstrates that the extracted VP can significantly improve the localization accuracy. In this paper, only the main VP (also called the primary VP) in continuous keyframes is integrated into the visual-inertial SLAM as global observation without drift error. The main VP is the intersection of parallel lines approximately along the direction of camera movement within the scope of the image. Through the main VP, we estimate the camera attitude to the current corridor. Furthermore, a series of methods is adopted to ensure the accuracy of the main VP (line removal and outlier VP filtering), thereby ensuring the accuracy of the relative pose of the camera calculated based on the VP. The camera attitude relative to the current corridor is the observation used to build the observation model.

The system drift error in visual-inertial SLAM is a cumulative value that changes continuously as the trajectory is traversed [9,10]. The back-end optimization of visual-inertial SLAM is a prerequisite to correct the cumulative errors and increase the state estimation accuracy [11,12]. Accordingly, this paper proposed novel non-linear optimization methods for a monocular camera and low-precision IMU, including the local optimization based on the sliding window and the global optimization based on the segmentation window. Moreover, the segmentation window is distinct from the sliding window in that it is self-contained. There is no special case in which an image frame is simultaneously assigned to two segmentation windows. In addition, the optimization methods are proposed based on the assumption that the drift error within the window is a random constant, and the system constrains the drift error of adjacent windows. Corridor detection is also performed. Additionally, scale drift occurs as a result of the inevitable pose estimation errors. By combining local optimization and global optimization, the location accuracy and robustness of the system can be significantly improved over other visual-inertial SLAM system.

To summarize, the main contributions of the proposed system are:

- Introducing an external observation without drift error into the visual-inertial SLAM, which is detected in the selected keyframe;
- Proposing the local optimization method based on the sliding window and the global optimization method based on the segmentation window to correct the system’s attitude estimation;
- External observation VP and PDR are obtained using the visual-inertial SLAM’s monocular camera and IMU, which wouldn’t increase the system’s hardware cost.

The study is organized as follows. The related work of the proposed method is discussed in Section 2. The main contexts are described in detail in Section 3, and the system overview is described in Section 3.1. A series of experiments and evaluation of the results are shown in Section 4. Finally, the paper is summarized in Section 5, and the future research direction of the work is also discussed in this Section.

## 2. Related Works

### 2.1. Visual-Inertial SLAM

The visual-inertial SLAM optimization methods can be classified into two categories: front-end optimization and back-end optimization. Non-linear optimization supplanted the traditional KF method, beginning with the parallel tracking and mapping (PTAM) method [13]. In comparison with the filter-based method, the optimal solution via iteration consumes more computing resources. As a result, sliding windows are frequently employed in a nonlinear system [13,14,15]. Shen et al. proposed a novel state estimator that incorporated both visual and inertial information. The state estimator adopted loop detection, relocalization, and global pose graph optimization to eliminate drift in visual-inertial SLAM’s back-end. Additionally, the visual-inertial alignment is proposed as a method for recovering the scale [16,17,18]. Karto SLAM decoupled sparse systems through the use of the non-iterative Cholesky matrix. The nodes and the edges represent the estimated pose and the constraints between nodes, respectively [19]. For the cartographer, global loop closure detection and subgraph optimization are combined to correct the cumulative errors [20]. In 2021, Chen et al. developed a back-end global map optimization method named global pose map optimization (GGO) after loop closure detection to reduce cumulative errors. The node is the pose of the keyframes selected in the front-end, and the constraint edge is the relative motion between the two pose nodes. DO-LFA adopted the nonlinear least-squares adjustment methods to obtain better results [21]. Kong et al. conduct two Gauss–Newton iterations alternately rather than jointly optimize all parameters simultaneously, avoiding the multidimensional Jacobian matrix. The proposed albedo-consistency bundle adjustment method combined the albedo model and the refined surface normal for optimization [22]. In VIR-SLAM, the ultra-wideband (UWB) factor ranging with the estimated anchor is used to correct the accumulated error by Cao et al. [1].

Due to the complementarity of the camera and IMU data, the cumulative drift error of the visual-inertial SLAM cannot be accurately corrected without loop closures. Therefore, numerous studies proposed to constrain the drift error of the system by introducing the external observation and measurement. In 2015, StructSLAM utilized the building structure lines as global orientation information to constraint the heading of the camera over time [23]. In addition, StructSLAM exploits the orientation information of the structure lines as a global constraint on the estimation, which means the error at each time step will be rectified globally. Therefore, the errors are not accumulated but bounded during the whole trajectory [24], integrating VP into the feature-based VIO to remove the angle drift, using a local optimization method based on sliding window. The basic odometry was a multi-state constraint Kalman filter (MSCKF) for vision-aided inertial navigation. To test the system’s performance, a global shutter fisheye camera was installed on the moving robot. Luo et al. incorporated the reprojection constraints and the relative poses constraints into the joint optimization algorithm in 2021. Besides, the double window structure can reduce scale drift [25]. Since the new frame’s pose is calculated by the state propagation, the error is accumulated over time in PALVO. The inevitable errors of pose estimation will also cause the scale drift when the estimated trajectory is long [26]. Based on PALVO, the Sim (3) relative pose is the constraint of pose graph optimization in reducing the error accumulation and scale drift. The global pose of all keyframes is fine-tuned by minimizing the reprojection error after the optimization [7]. In 2021, Zhao et al. proposed super odometry, a framework that combined light detection and ranging (LiDAR), a camera, and the IMU sensor. In the super odometry, the estimated pose of visual-inertial odometry and laser-inertial odometry are IMU bias’s constraint. The IMU odometry constrains the other odometry [21,27] as well. Other constraints are imported into the laser-visual-inertial system, such as thermal-inertial [28], and leg odometry [29]. Cao et al. introduced a static ultra-wideband (UWB) anchor as a global positioning system to improve the localization accuracy [1]. Before the first turn, there is no rotation information for pose estimation. Therefore, the IMU and wheel encoder measurements are fused by the bidirectional trajectory computation method, and the wheel encoder is regarded as an extra odometry [30]. In 2020, Shan et al. proposed a tight-couple LiDAR odometry system, containing LiDAR, IMU, and GPS. In GLO-SLAM, the reliable GPS is the global optimization measurement to eliminate the accumulative errors [31].

It is feasible to reduce the gyroscope’s accumulated drift error with the newly introduced external observation. The obvious feature of the methods mentioned above is the addition of new sensors, which may incur a high cost, multi-sensor information fusion, etc. Therefore, it is unnecessary to consider the potential issues caused by the new sensor if the existing camera and IMU can provide external observation in the visual-inertial SLAM.

### 2.2. Vanishing Point

According to the perspective principle, a set of parallel lines in the 3D object space will intersect at a point known as VP after projection [32]. In 2021, Khac et al. proposed a robust motion-based road VP detection method (R-VP detection method) for real-world driving scenes. In the study, the proposed method that consists of stable motion detection, stationary point-based motion vector selection, and angle-based RANSAC voting achieves high accuracy and robustness in the Jiqing Expressway dataset [33]. In 2018, an accurate and efficient road vanishing point detection scheme based on the v-disparity and visual odometry techniques was proposed. In the system, the v-disparity map could significantly reduce the searching space for vanishing point. Another important value of the VP is the relative pose estimation of the camera to the vehicle, the corridor, or other objects in real world. The camera orientation can be estimated using the geometric correlation between a line, a VP, and the camera orientation [8]. In 2018, a camera orientation estimation method based on 3-line RANSAC using motion vector in a vehicle driving straight ahead was proposed. The study utilized feature extraction and matching to estimate the *Z*-axis vanishing point. Note that, the VP was the intersection of classified lines, which were determined by a 3-line RANSAC algorithm. Li et al. focused on using a convolutional neural network to predict the VP in fisheye images, where VP detection is regarded as a regression problem [34]. The VP can also be used to estimate the camera parameters [32,35,36]. Kocur et al. proposed a traffic surveillance camera calibration method based on vanishing points pairs associated with vehicles in the traffic surveillance footage [35]. A novel pretrained CNN was proposed to detect pairs of the VP, which output heatmaps. From the detected pairs of vanishing points for multiple vehicles in a scene, the study also established the scene geometry by estimating the focal length of the camera and the orientation of the road plane.

Due to the good characteristics of VP, it has been used for lane line detection in automatic driving, camera pose estimation, and camera parameter calibration as mentioned above [36,37,38]. There is almost no research on applying the VP as a local or global observation to the visual-inertial SLAM. Therefore, this study integrates the VP into the odometry-aided visual-inertial SLAM to correct the cumulative drift error.

## 3. The Main Context

### 3.1. System Overview

**Figure 1:**
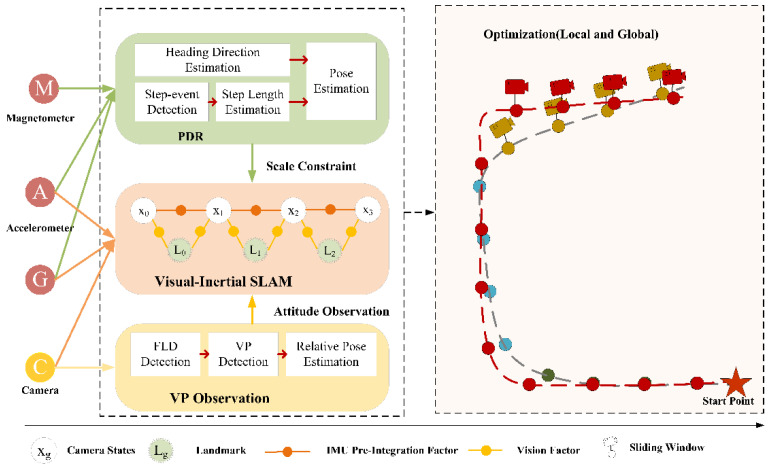
The system overview.

Figure 1 depicts a general overview of the proposed system. The system takes synchronized IMU data and monocular images as input, and outputs the camera poses for each keyframe. The keyframes are chosen in the vision processing the front-end based on the tracked number of feature points and the parallax change between adjacent image frames. Calculating PDR introduces parallax processes, which estimate the camera’s attitude and location, respectively [39]. Due to the scale drift error in the original visual-inertial SLAM, the proposed system incorporates the PDR observation as a scale constraint. In Figure 1, the circles in Optimization (Local and Global) represent various pose graph points. Additionally, each colored pose graph point corresponds to a distinct segmentation window. A blue dashed line represents the pose graph prior to correction, and a red dashed line represents the corrected pose graph.

In comparison with other external observations in an indoor environment, VP detection does not require continuity. As a result, the detection frequency flexibility can be adjusted to meet the computing power requirements. In this case, the system only detects the VP in keyframes to estimate the relative pose of the camera to the current corridor. Cumulative drift error exists in the estimated camera’s pose. Thus, the external observation VP without drift error is imported into the back-end optimization of the proposed system to correct the accumulated drift error mentioned previously.

The proposed back-end optimization methods include both local optimization and global optimization. The local optimization and global optimization are based on the sliding window and the segmentation window, respectively. The window size is adjustable. We ignore the drift error of a single frame and optimize the frames in the window fully. For local optimization, the least square method is used to estimate the only one offset in the window. In contrast to local optimization, global optimization is directed at all segmentation windows that are traversed. As the segmentation window’s amount increases, the number of variables to be estimated increase proportionately. Each time someone passes a new corridor, the preceding corridors are optimized. Section 3.2, Section 3.3 and Section 3.4 contain more details. The proposed system’s joint optimization is a critical insight.

### 3.2. Scale Drift Correction Based on PDR

Scale drift error exists in the visual-inertial SLAM because scale information is provided by the pre-integration of IMU in adjacent IMU moments [40]. Thus, the local constraints of the IMU pre-integration factor can be formulated by interpolating the visual-inertial odometry and pure inertial odometry (PDR). The IMU residuals $e_{I_{i}I_{j\;}}$ in consecutive IMU frames $I_{i}$ and $I_{j}$ are written as Equation (1):

(1) $$e_{I_{i}I_{j\;}}=\left[\begin{array}{c} {\hat{\alpha }}_{I_{j}}^{I_{i}}\\ {\hat{\beta }}_{I_{j}}^{I_{i}}\\ {\hat{r}}_{I_{j}}^{I_{i}} \end{array}\right]-\left[\begin{array}{c} R_{w}^{I_{i}}\left(p_{I_{j}}^{w}-p_{I_{i}}^{w}+\frac{1}{2}g^{w}\Delta t^{2}-v_{I_{i}}^{w}\Delta t\right)\\ R_{w}^{I_{i}}\left(v_{I_{j}}^{w}+g\Delta t-v_{I_{i}}^{w}\right)\\ {\left(q_{I_{i}}^{w}\right)}^{-1}⊗q_{I_{j}}^{w} \end{array}\right]$$

where $\left({\hat{\alpha }}_{I_{j}}^{I_{i}},{\hat{\beta }}_{I_{j}}^{I_{i}},{\hat{r}}_{I_{j}}^{I_{i}}\right)$ is the relative motion measurement between consecutive IMU frames $I_{i}$ and $I_{j}$. $p_{I_{j}}^{w}$, $v_{I_{j}}^{w}$*,* $q_{I_{j}}^{w}$ are the translation, velocity, and rotation from the IMU frame to world frame, which can be obtained by the VIO and the PDR.

Since the IMU pre-integration measurements are constrained by the PDR observation, it is possible to obtain multiple IMU pre-integration residuals simultaneously. The pedestrian’s location and pedestrian’s velocity time t contain components in three directions, including x, y, and z. The velocity in the z-axis is assumed to be zero. PDR is calculated by pure inertial odometry. The residuals are weighted by the appropriate covariance matrix $\mu_{I_{i}I_{j}}$, which relies on the reliability of the observation [27,41]. For instance, when the light condition changes from dark to light or from light to dark, the visual-inertial SLAM exhibits a larger scale drift, whereas the PDR observation has a large weight. The optimization problem for each new IMU frame consists of VIO-IMU terms, PDR-IMU terms and marginalization prior $E_{prior}$.

(2) $$E=\underset{\left(I_{i},I_{j}\right)\in ∁}{\sum }{\left(e_{I_{i}I_{j}}^{VIO}\right)}^{T}{\left(\mu_{I_{i}I_{j}}^{VIO}\right)}^{-1}e_{I_{i}I_{j}}^{VIO}+{\left(e_{I_{i}I_{j}}^{PDR}\right)}^{T}{\left(\mu_{I_{i}I_{j}}^{PDR}\right)}^{-1}e_{I_{i}I_{j}}^{PDR}+E_{prior}$$

**Figure 2:**
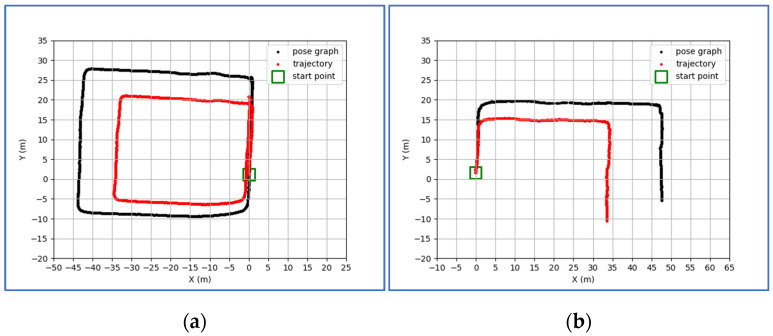
The comparison between the pose graph and scale-restored trajectory. (**a**,**b**) reflect the effect of PDR on correcting the scale of various length trajectories.

The principle of using PDR to correct the visual-inertial SLAM is relatively simple. The comparison results of two groups are shown on Figure 2a,b. The tests are conducted on the self-collected dataset, where the length of the corridor is 33.6 m, the width is 25.7 m. The results indicate that PDR can correct the scale of visual-inertial SLAM truly and effectively.

### 3.3. The Camera Pose Estimator Based on VP

In an indoor environment, particularly one characterized by a long straight corridor, the VP is a useful feature for characterizing the environment. We define the dominant direction as the direction in which the camera is moving. In the practical implementation, only the main VP is detected in the keyframe.

**Figure 3:**
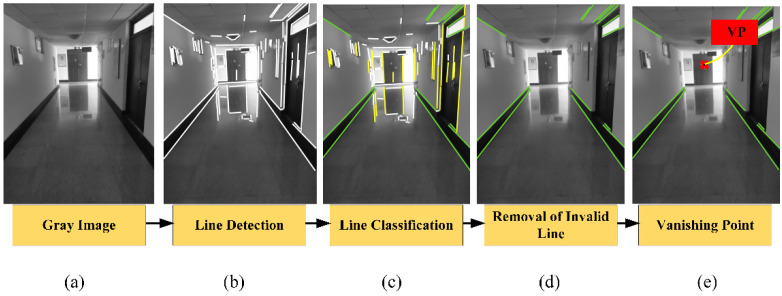
The detection steps of VP. Lines in green are remained.

The VP extraction can be simply summarized in four steps, as illustrated in Figure 3. The image captured by the monocular camera exists radial distortion and lateral distortion. The RGB image captured by the monocular camera is converted into the gray image after the image distortion. All potential lines are detected in the line detection step. In real scenes, a cluster of parallel lines may include many spurious parallel lines on the 2D image because of the characteristics of artificial structures. Moreover, there are lines parallel to the dominant direction and lines perpendicular to the dominant direction. As a result, the unqualified lines are removed in Figure 3d. Finally, the intersection of the remained lines is the VP, which is colored in red in Figure 3e.

The matrix vector equation of the remained lines is defined as follows:

(3) Mp=c

(4) $$\left[\begin{array}{c} \begin{array}{cc} a_{1} & b_{1}\\ a_{2} & b_{2} \end{array}\\ \begin{array}{cc} \vdots & \vdots \end{array}\\ \begin{array}{cc} a_{\chi } & b_{\chi } \end{array} \end{array}\right]\left[\begin{array}{c} p_{u}\\ p_{v} \end{array}\right]=\left[\begin{array}{c} c_{1}\\ c_{2}\\ \begin{array}{c} \vdots \\ c_{s} \end{array} \end{array}\right]$$

where χ is the amount of the remained lines. $\left(a_{l},b_{l},c_{l}\right)$ are the coefficients of the (l)th equation in Equation (3), and *M* is a matrix composed of $a_{l}$ and $b_{l}$. $\left(p_{u},p_{v}\right)$ represents the location of the VP in image coordinates. The estimated VP, $\hat{p}=({\hat{p}}_{u},{\hat{p}}_{v})$, is calculated with the least square methods using a Penrose pseudo-inverse matrix as Equation (5):

(5) $$\hat{p}={\left(M^{T}M\right)}^{-1}M^{T}$$

On the other hand, the camera’s orientation with respect to the scene is related to the camera intrinsic parameters and the location of the VP in three orientations, including x, y, z. The relationship between camera orientation R, intrinsic parameters $k_{cal}$, and the location of the VP $Loc_{p}$ is denoted as follows:

(6) $$Loc_{p}=k_{cal}R$$

where $Loc_{p}$ in homogenous pixel coordinates is defined as Equation (7):

(7) $$Loc_{p}=\left[\begin{array}{ccc} {\hat{p}}_{xu} & {\hat{p}}_{yu} & {\hat{p}}_{zu}\\ {\hat{p}}_{xv} & {\hat{p}}_{yv} & {\hat{p}}_{zv}\\ 1 & 1 & 1 \end{array}\right]$$

The calibration matrix is defined as Equation (8). In Equation (8), the focal length $\left(f_{x},f_{y}\right)$ defines the distance between the center of the lens and the film while taking a focused image of an infinitely far object. The intersection of the image plane and the optical axis of the camera is the principal point $\left(c_{x},c_{y}\right)$. When testing the system, two different models of smartphones are used. The calibration parameters of these two smartphones are summarized in Table 1.

(8) $$k_{cal}=\left[\begin{array}{ccc} f_{x} & 0 & c_{x}\\ 0 & f_{y} & c_{y}\\ 0 & 0 & 1 \end{array}\right]$$

For the known coordinate system, the rotation matrix R of the camera expressed in Euler angles is defined as follows:

(9) $$R=\left[\begin{array}{ccc} \cos\varphi cos\emptyset & cos\varphi sin\emptyset sin\theta -sin\varphi cos\theta & cos\varphi sin\emptyset cos\theta +sin\emptyset sin\theta \\ sin\varphi cos\emptyset & sin\varphi sin\emptyset sin\theta +cos\varphi cos\theta & sin\varphi sin\emptyset cos\theta -cos\varphi sin\theta \\ -sin\emptyset & cos\emptyset sin\theta & cos\emptyset cos\theta \end{array}\right]$$

where φ, ∅, and θ is yaw, pitch, and roll, respectively.

In the actual test environment, the system only uses the main VP. According to Equations (3)–(8), the relative pose of the camera to the current corridor meets the constraint:

(10) $$f_{x}\left(cos\varphi sin\emptyset cos\theta +sin\emptyset sin\theta \right)+c_{x}cos\emptyset cos\theta ={\hat{p}}_{zu}\;f_{y}\left(sin\varphi sin\emptyset cos\theta -cos\varphi sin\theta \right)+c_{y}cos\emptyset cos\theta ={\hat{p}}_{zv}\;cos\emptyset cos\theta =1$$

### 3.4. The Local Optimization Method Based on the Sliding Window

**Figure 4:**
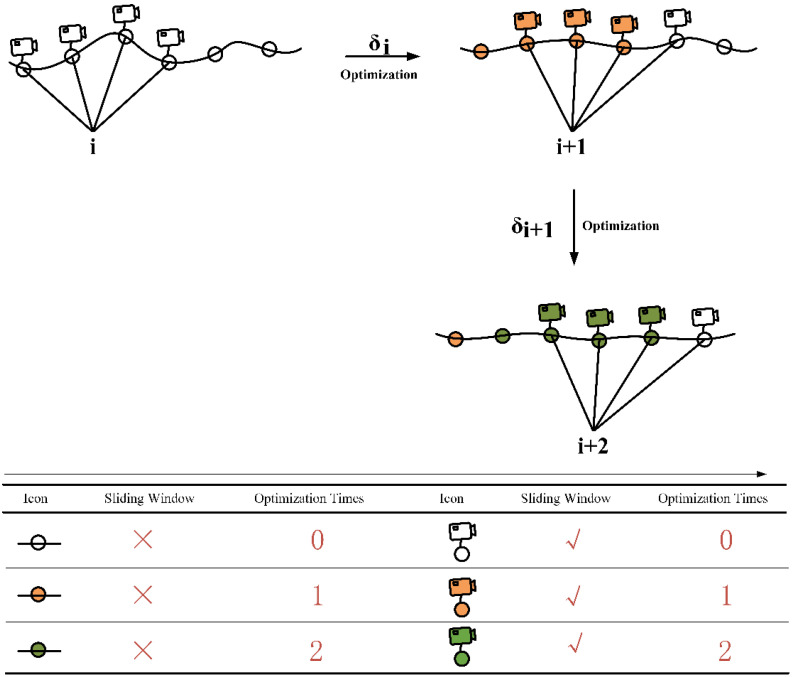
The process of the local optimization based on the sliding window.

The local optimization of the pose graph is proposed based on the sliding window. Figure 4 illustrates the process of the local optimization. s frames are maintained in each sliding window, and s can be adjusted to meet the various actual requirements. For the test experiments of the ADVIO dataset and the self-collected dataset, s=4. For the initial n(n≤s) keyframes of a new corridor, the local optimization is also performed to avoid the large initial pose errors. As mentioned previously in Section 3.2, the estimated pose of the camera by the VP is relative to the current corridor. Thus, the sliding window cannot contain keyframes from two corridors simultaneously. How to judge the different corridors? The system assumes that the yaw $\varphi_{VI}$ estimated by the visual-inertial SLAM in the same corridor remains within a certain range. That is when the change amount of $\varphi_{VI}$ in consecutive keyframes exceed the set threshold ϵ, the corridor frame is changed [37,42,43]. When a new corridor is detected, the last corridor’s local optimization is stopped, and the current corridor’s local optimization is continued.

The system assumes that the drift error $\delta_{i}$ in the (i)th window is a constant, which correspond to the drift error of s keyframes in the (i)th window. Additionally, the drift error in the continuously sliding window satisfies the constraint, where $\lambda_{1}$ and $\lambda_{2}$ are scale factors:

(11) $$\lambda_{1}\delta_{i}\leq \delta_{i+1\;}\leq \lambda_{2}\delta_{i}$$

The definition of the reference frame is denoted as follows: the world frame is denoted as W; the IMU frame is denoted as I; the camera frame is denoted as C, and the corridor frame is denoted as B. The transformation matrix $R_{W}^{B}\in SE\left(3\right)$ transforms the vector from the frame W to frame B, such as the vector $\varphi_{VI}$. To achieve observation Equation (1), the estimated pose ($p_{j}^{W},q_{j}^{W}$) by the visual-inertial SLAM in the (j)th keyframe of the (i)th sliding window is transformed to the corridor frame, the transformation matrix is $R_{W}^{B}$:

(12) $$y\left(j\right)=R_{W}^{B}\left(x\left(j\right)-\delta_{j}\right),\;\;\;\;\;\;\;j=1,2,\ldots ,s$$

where y(j) is the estimated relative pose of the camera in corridor frame, and the x(j) is the estimated pose of the camera in visual-inertial SLAM. $\delta_{j}$ is estimated by the nonlinear least-squares method. Then the pose of the camera $\left({\hat{p}}_{j}^{w},{\hat{q}}_{j}^{w}\right)$ in the world frame of the (j)th keyframe is updated.

(13) $${\hat{p}}_{j}^{w}=p_{j-1}^{w}+\hat{v}\Delta t\;{\hat{q}}_{j}^{w}=q_{j}^{w}+R_{B}^{W}\delta_{j}$$

where $\hat{v}=\left({\hat{v}}_{x},{\hat{v}}_{y},{\hat{v}}_{z}\right)$ represents the updated speed components of x, y, z axis, respectively.

### 3.5. The Global Optimization Method Based on the Segmentation Window

**Figure 5:**
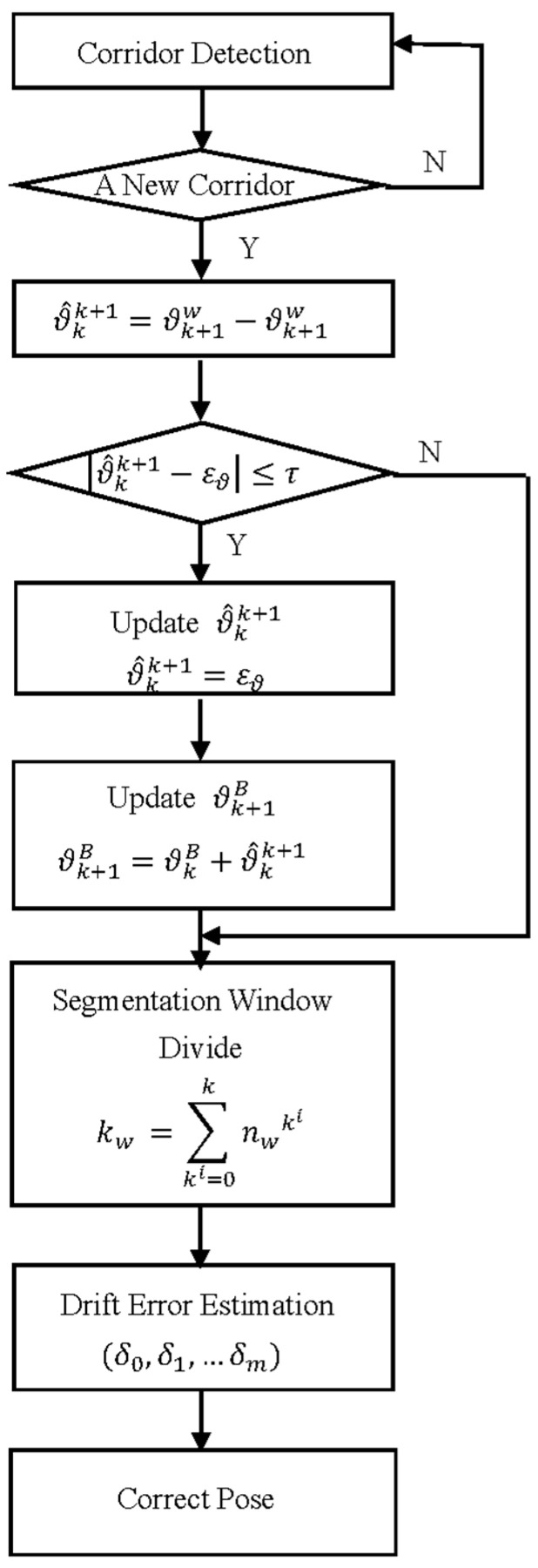
The flow chart of global optimization is based on the segmentation window.

The local optimization based on the sliding window aims at continuously optimizing the drift error in the window until the estimation of the entire trajectory is completed. Global optimization, in contrast to local optimization, occurs after the corridor is updated. Global optimization is an optimizer for all previously traversed corridors. Once the trajectory is completely estimated, the last global optimization can be performed. Figure 5 illustrates the optimization process.

The variables used in global optimization are as follows: the estimated pose of the camera in the world frame is $\vartheta_{k}^{w}$, where k denotes the number of the corridor. The angle between the (k)th corridor and the (k+1)th corridor is ${\hat{\vartheta }}_{k}^{k+1}$. The parameter $\varepsilon_{\vartheta }$ in our system is set as an integer multiple of 45°. The set threshold τ is 1°. As mentioned above, the global optimization is based on the segmentation window. The size of the segmentation is set as $s_{w}$. The total number of the segmentation window that must be optimized in the global optimization is $n_{w}$, which also represents the number of variables to be estimated.

The camera pose calculated based on the VP is its relative pose to the current corridor in which it is located. Therefore, it cannot reflect the relationship between the estimated camera pose under the current and preceding corridors, nor can it reflect the relationship between the estimated camera pose under the current corridor and the initial corridor. The angle between adjacent corridors was corrected in response to the proposed issue. According to the corridor detection method, if the angle ${\hat{\vartheta }}_{k}^{k+1}$ of the adjacent corridor [k,k+1] is nearly $\varepsilon_{\vartheta }$, $\vartheta_{k+1}=\varepsilon_{\vartheta }$, then all the relative poses of the camera to the current corridor are updated to the initial corridor’s pose.

The segmentation size of the global optimization $s_{w}$ is a constant. The distinction between the global optimization and local optimization is that the global optimization is a multivariate estimation problem, whereas local optimization is not. That is, the drift error will be estimated simultaneously for multiple segmentation windows. Additionally, the drift error in the segmentation window is a random constant. The number of the segmentation window in the (k)th corridor can be calculated as follows:

(14) $$n_{w}{}^{k}=\{\begin{array}{c} int(f_{k}/s_{w}),\;if\;(f_{k})\%s_{w}=0\\ int(f_{k}/s_{w})+1,\;else \end{array}$$

where $f_{k}$ is the frame number of the (k)th corridor.

Note that the drift error of the (m)th segmentation window is calculated as follows:

(15) $$y\left(n_{w}^{m}\right)=R_{W}^{B}\left(x\left(n_{w}^{m}\right)-\overset{n_{w}^{m}-1}{\underset{m=0}{\sum }}\delta_{m}\right)$$

## 4. Experiment and Result

The entire system was built on the ROS system in Ubuntu 16.04. A series of experiments were carried out on a desktop equipped with an Intel Core i7-8700 CPU (3.20 GHz), 16.0 GB of RAM, and an NVIDIA GeForce GTX1650 with 2 GB of graphic memory. In addition, both the public ADVIO dataset and the self-collected dataset were used to test the proposed system’s reliability.

### 4.1. Dataset

**Figure 6:**
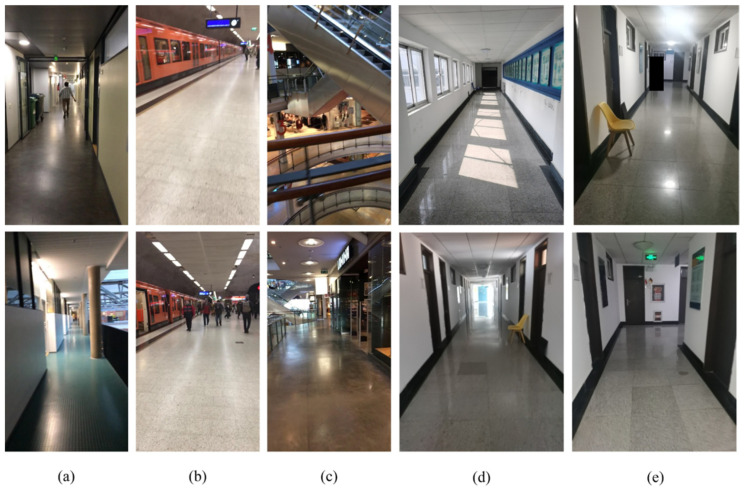
The various scenes of the ADVIO dataset and the self-collected dataset. (**a**–**c**) represents the office building, subway, and mall scenes of the public ADVIO dataset, respectively. (**d**,**e**) shows the office environment of the self-collected dataset. (**d**) is collected during the day, and (**e**) is collected during the night.

The performance of the proposed system was evaluated on the public ADVIO dataset [44] and the self-collected dataset. The ADVIO dataset is widely used in visual-inertial SLAM, and the data collection equipment contains iPhone, Tango, Google’s ARCore, and a camera. The indoor scenes of the ADVIO dataset include a subway, mall, and office building scene, as illustrated in Figure 6a–c. The visual-inertial SLAM preforms worse in the dynamic environment compared with the static environment according to [31,45,46]. Additionally, if the trajectory contains a rotation angle close to an integer multiple of 45°, the performance of the global optimization in correcting the drift error is obvious. After comprehensively considering the differences in scenarios, data characteristics, and algorithm verification requirements, the public Advio_17 (office scene) was selected for algorithm verification. We used a calibrated smartphone to record a series of data sequences in Qingdao. In addition, another smartphone recorded the collection process in the video. In practice, the manual fixation points were aligned and the relative position of those fixed points were precise. The data streams (image data and IMU data) from the smartphone were synchronized using the UTC time. The collector walked down the corridor at an average peace of 1.4 to 1.5 m/s, slightly shaking the smartphone. The ground truth was calculated by implementing the purely inertial odometry proposed by [47]. Because the data were collected from the same height, the height of the ground truth in the fixed position is a constant. Figure 6d,e presents the self-collected scenes. The following summarizes the characters of the self-collected dataset:

(1) Different layer environment: the scene on the fourth floor is simpler, while the scene on the first floor is significantly more complex and contains more semantic objects;
(2) Different light conditions: day and night; light and dark;
(3) Different movement distance: short, medium, and long.

**Figure 7:**
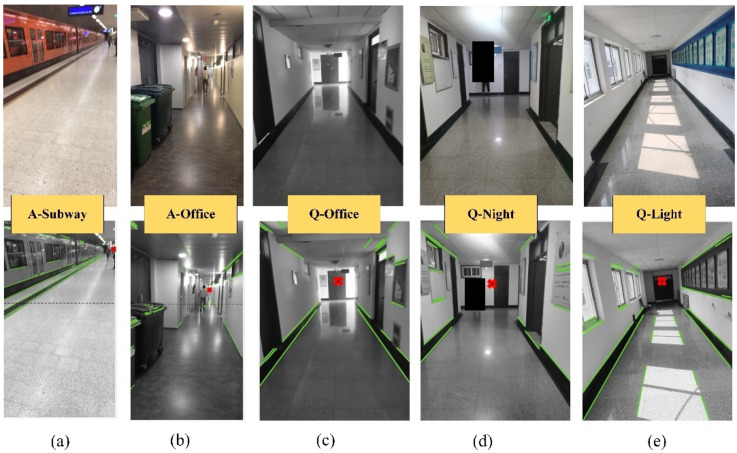
Row1: the original RGB images; Row2: the detection results of line and VP.

The lines and VP detection results for each image in the public ADVIO dataset (A-Subway and A-Office) and self-collected dataset (Q-Office, Q-Night, Q-Light) are labeled in Figure 7. We also performed VP detection in a mall scene, but the detection result is not ideal. Therefore, only the VP detection results of ADVIO dataset’s subway and office scenes are shown on Figure 7.

### 4.2. Performance Evaluation of the Proposed System

The metrics absolute trajectory error (ATE) and relative pose error (RPE) were used to evaluate the performance of the proposed system. Metric ATE stands for the trajectory’s global consistency, whereas metric RPE reflects the translational and rotational drift.

Table 2 and Table 3 present quantitative comparison results of the self-collected dataset (01, 02) and the public ADVIO dataset (Advio_17). The mean errors are concerned to indicate the proposed system’s robustness and stability. The proposed system’s percent improvement over the original visual-inertial SLAM is represented. The improvement values of the mean error η in Table 2 and Table 3 are calculated as follows:

(16) $$\eta =\frac{ο-\xi }{ο}\times 100\%$$

where ο represents the mean error of the original visual-inertial SLAM and ξ represents the mean error of the proposed system. We tested several visual-inertial slams including VINS-Mono [48], VINS-Fusion [49] and ORB-SLAM2 [19]. VINS-Mono was more stable than other tested slams for the self-collected dataset and the public ADVIO dataset; therefore, we chose VINS-Mono as the original visual-inertial SLAM.

**Figure 8:**
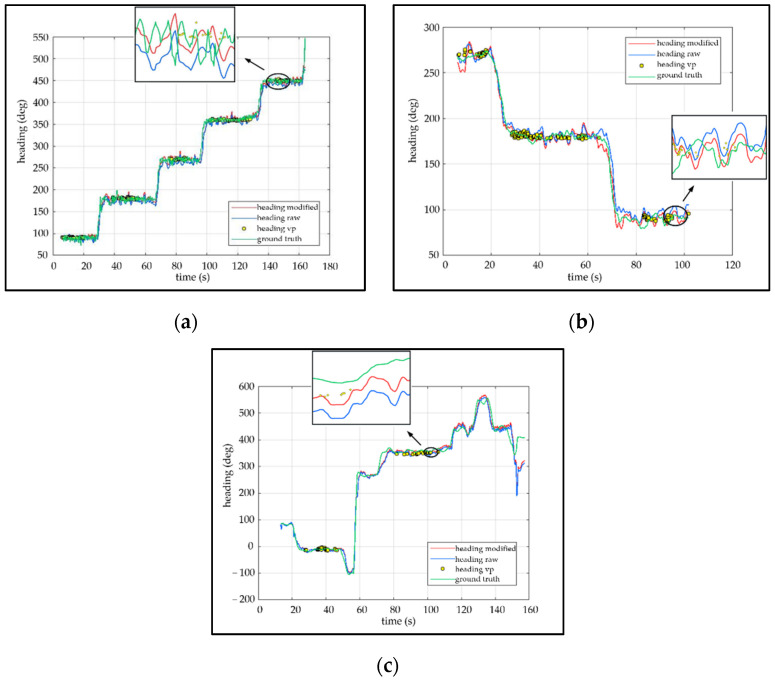
The attitude comparison between modified heading, raw heading, estimated heading based on VP and ground truth. (**a**–**c**) shows the attitude comparison of the self-collected dataset 01, self-collected dataset 02, and public dataset Advio_17, respectively.

**Figure 9:**
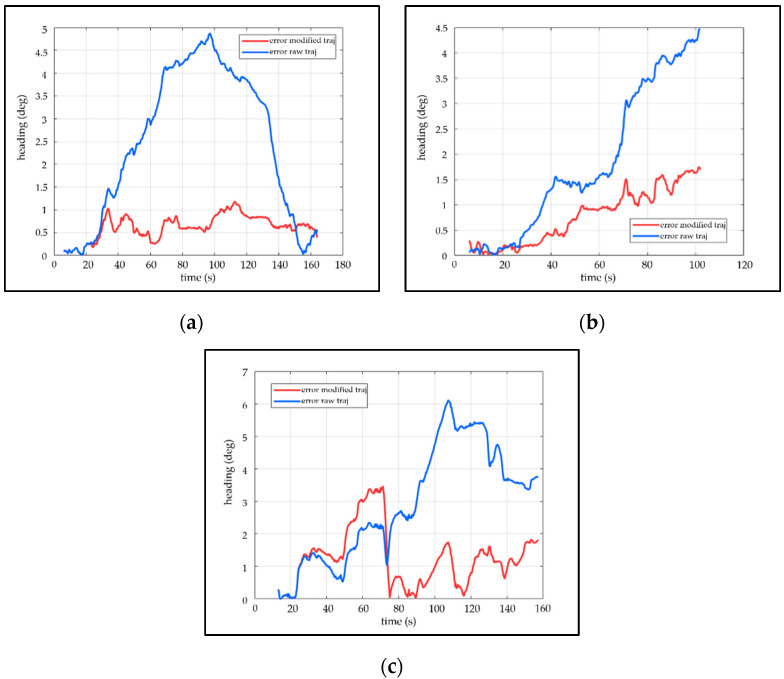
The attitude error comparison of the modified attitude and the original attitude. (**a**–**c**) shows the attitude error comparison of the self-collected dataset 01, self-collected dataset 02, and public dataset Advio_17, respectively.

The Euler angle stands for one type of camera ego-motion, containing yaw, pitch and roll. There is only the yaw comparison in Figure 8. To facilitate comparisons, the partial results are enlarged and displayed in the form of small windows. The small window demonstrates that the corrected posture is closer to the ground truth. After a long period of accumulated drift, the attitude error of the original visual-inertial SLAM is larger than the modified, as illustrated in Figure 9. In Figure 9a,c, the drift error of the original visual-inertial SLAM increases initially and then decreases. The reasons may be error cancellation for circular motion. While the drift error in Figure 9a,c is decreasing, the average error of the uncorrected angle still exceeds the corrected angle by the VP, as shown in Table 2.

As shown in Table 3, the performance of the original visual-inertial SLAM can be improved by an order of magnitude in the majority of application environments. For the self-collected dataset, the accumulated drift error of the proposed system is less than 1 m, while the accumulated drift error of the original visual-inertial SLAM exceeds 1.5 m. In 01 and 02, the drift error is corrected by 75.12% and 58.12%, respectively. The results in Table 3 indicate that the robustness and stability of the SLAM system can be improved by the proposed system in application scenes.

**Figure 10:**
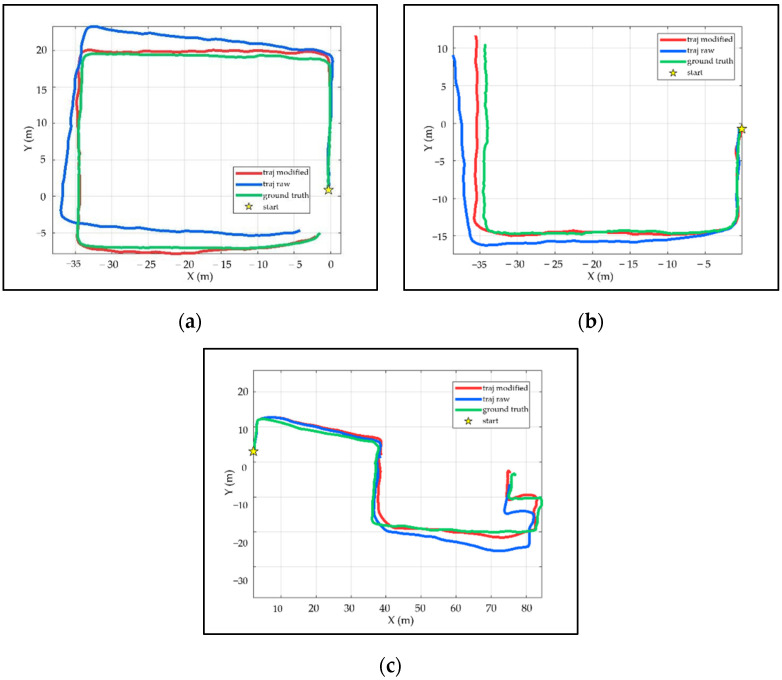
The trajectory comparison between modified trajectory, raw trajectory, and ground truth. (**a**–**c**) shows the trajectory comparison of the self-collected dataset 01, self-collected dataset 02, and public dataset Advio_17, respectively.

The larger the accumulated drift error, the more the estimated trajectory deviates from the ground truth on the pose graph. Figure 10 illustrates intuitively the comparison of the modified trajectory, raw trajectory and ground truth, which corresponds to traj modified, traj raw and ground truth in Figure 10, respectively. In Figure 10, the raw trajectory deviates, increasing from the ground truth over time, whereas the modified trajectory approaches the ground truth more closely. Note that, PDR corrects both the raw trajectory and the modified trajectory. The overall length of the trajectory in Figure 10 varies due to the different turn times.

## 5. Conclusions and Discussion

To correct the accumulated drift error of the visual-inertial SLAM system, an enhanced pedestrian visual-inertial SLAM system aided with the vanishing point in an indoor environment is proposed. The proposed system is characterized by the enhanced VP-based global observation for pose optimization taking advantage of the corridor detection. Compared with the conventional VP-based local observation, the global observation refers to the calculated relative pose of the camera to the initially detected corridor. The system assumes that the drift error in the window is a random constant. Based on the assumption, we propose the local optimization based on the sliding window and the global optimization based on the segmentation window. Note that global optimization is a multivariate state estimation problem. Corridor detection is performed to calculate the relative pose of the camera to the initial corridor and divide the window more reasonably. In addition, PDR is also integrated into the proposed system as inertial odometry to correct scale error. The system uses a monocular camera and a low-cost IMU, which can be integrated into smartphones or other commonly used portable devices easily.

Besides evaluation on the public ADVIO dataset, the experimental tests were performed with the self-collected dataset using an off-the-shelf smartphone to verify the proposed visual-inertial SLAM performance in real-world applications of pedestrian positioning. The experimental results are evaluated by the metrics absolute trajectory error and relative pose error. These tests also confirm that it is practicable to use our proposed optimization method to correct the drift error of the system and improve the location accuracy in an indoor environment.

Now that the proposed system is verified to improve the accuracy of a single trajectory, how to fuse the information from multiple trajectories to construct a scene map, which is also known as crowdsourced mapping, is a complex topic we have been researching. The crowdsourced map is applicable for positioning and navigation. In addition, other sensor information (GNSS, WIFI, Bluetooth, etc.) can be integrated into the proposed system for data fusion and crowdsourced mapping. At the same time, other practical applications should be challenged, such as the positioning of vehicles in underground parking lots.

## Figures and Tables

**Table 1:** The calibration parameters of two different types of smartphones.

Parameters		Smartphone 01	Smartphone 02
Intrinsic	$f_{x}$	1082.4	392.92
	$f_{y}$	1084.4	392.82
	$c_{x}$	364.6778	186.19
	$c_{y}$	643.3080	247.80
Distortion Coefficient	$k_{1}$	0.0366	−0.0028
	$k_{2}$	0.0803	0.002
	$p_{1}$	0.000783	0
	$p_{2}$	−0.0002	0
Extrinsic		$\left[\begin{array}{cccc} 0.999976 & -0.004079 & -0.005539 & -0.008977\\ -0.004066 & -0.999989 & 0.002323 & 0.075570\\ -0.005548 & -0.002300 & -0.999981 & -0.005545\\ 0 & 0 & 0 & 1 \end{array}\right]$	

**Table 2:** Absolute attitude mean error [**°**] of the proposed method against the original visual-inertial SLAM.

Sequence	Source	Original Visual-Inertial SLAM [°]	Our Proposed Method [°]	Improvements [%]
01	self-collected	6.4521	1.3709	78.75
02	self-collected	4.1428	0.2807	93.22
Advio_17	public	6.2999	1.6886	73.20

**Table 3:** Absolute trajectory mean error [M] of the proposed method against the original visual-inertial SLAM.

Sequence	Source	Original Visual-Inertial SLAM [m]	Our Proposed Method [m]	Improvements [%]
01	self-collected	2.5521	0.63486	75.12
02	self-collected	1.8314	0.76699	58.12
Advio_17	public	2.8541	1.2832	55.04

## Data Availability

Not applicable.
